# Methyl 4-(3-eth­oxy-4-hydroxy­phen­yl)-6-methyl-2-oxo-1,2,3,4-tetra­hydro­pyrimidine-5-carboxyl­ate monohydrate

**DOI:** 10.1107/S160053680902769X

**Published:** 2009-07-18

**Authors:** M. Thenmozhi, T. Kavitha, V. S. V. Satyanarayana, V. Vijayakumar, M. N. Ponnuswamy

**Affiliations:** a Centre of Advanced Study in Crystallography and Biophysics, University of Madras, Guindy Campus, Chennai 600 025, India; b Organic Chemistry Division, School of Science and Humanities, VIT University, Vellore 632 014, India

## Abstract

In the title compound, C_15_H_18_N_2_O_5_·H_2_O, the pyrimidine ring adopts a flattened-boat conformation. The eth­oxy group attached to the benzene ring is in an extended conformation. The oxopyrimidine mol­ecules are linked into centrosymmetric *R*_2_^2^(20) dimers by O—H⋯O hydrogen bonds. The dimers are linked by N—H⋯O hydrogen bonds, forming a two-dimensional network parallel to the *bc* plane. Adjacent networks are cross-linked *via* N—H⋯O and O—H⋯O hydrogen bonds involving the water mol­ecules.

## Related literature

For the biological properties of pyrimidine compounds, see: Kidwai *et al.* (2003 ▶). For C=O bond-length data, see: Litvinov *et al.* (1992 ▶). For hybridization, see: Beddoes *et al.* (1986 ▶). For ring conformational analysis, see: Cremer & Pople (1975 ▶); Nardelli (1983 ▶). For graph-set analysis, see: Bernstein *et al.* (1995 ▶).
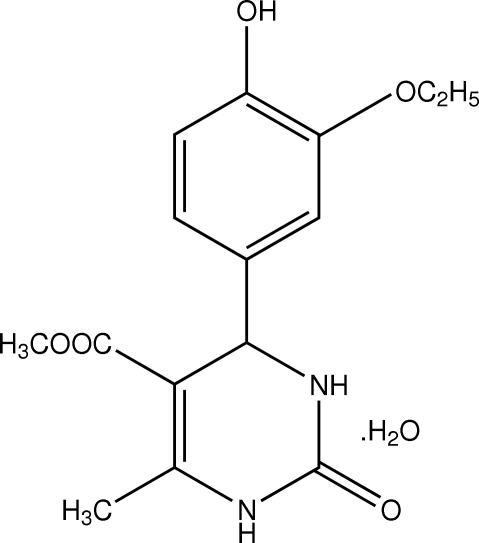


## Experimental

### 

#### Crystal data

C_15_H_18_N_2_O_5_·H_2_O
*M* *_r_* = 324.33
Monoclinic, 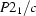

*a* = 11.4927 (6) Å
*b* = 15.3756 (8) Å
*c* = 8.9240 (5) Å
β = 95.932 (2)°
*V* = 1568.49 (15) Å^3^
*Z* = 4
Mo *K*α radiation
μ = 0.11 mm^−1^
*T* = 293 K
0.25 × 0.20 × 0.20 mm

#### Data collection

Bruker Kappa APEXII area-detector diffractometer
Absorption correction: multi-scan (*SADABS*; Sheldrick, 2001 ▶) *T*_min_ = 0.977, *T*_max_ = 0.981
20671 measured reflections
4719 independent reflections
3292 reflections with *I* > 2σ(*I*)
*R*_int_ = 0.025

#### Refinement

*R*[*F* ^2^ > 2σ(*F* ^2^)] = 0.048
*wR*(*F* ^2^) = 0.140
*S* = 1.04
4719 reflections
231 parameters
H atoms treated by a mixture of independent and constrained refinement
Δρ_max_ = 0.31 e Å^−3^
Δρ_min_ = −0.25 e Å^−3^

### 

Data collection: *APEX2* (Bruker, 2004 ▶); cell refinement: *SAINT* (Bruker, 2004 ▶); data reduction: *SAINT*; program(s) used to solve structure: *SIR92* (Altomare *et al.*, 1999 ▶); program(s) used to refine structure: *SHELXS97* (Sheldrick, 2008 ▶); molecular graphics: *ORTEP-3* (Farrugia, 1997 ▶); software used to prepare material for publication: *SHELXL97* (Sheldrick, 2008 ▶) and *PLATON* (Spek, 2009 ▶).

## Supplementary Material

Crystal structure: contains datablocks global, I. DOI: 10.1107/S160053680902769X/ci2843sup1.cif

Structure factors: contains datablocks I. DOI: 10.1107/S160053680902769X/ci2843Isup2.hkl

Additional supplementary materials:  crystallographic information; 3D view; checkCIF report

## Figures and Tables

**Table 1:** Hydrogen-bond geometry (Å, °)

*D*—H⋯*A*	*D*—H	H⋯*A*	*D*⋯*A*	*D*—H⋯*A*
N1—H1⋯O6	0.87 (2)	2.02 (2)	2.890 (2)	172 (2)
N3—H3⋯O1^i^	0.85 (2)	2.28 (2)	3.031 (2)	147 (2)
O2—H2⋯O5^ii^	0.90 (2)	2.07 (2)	2.810 (2)	139 (2)
O6—H6*A*⋯O1^iii^	0.92 (3)	1.87 (3)	2.777 (2)	170 (2)
O6—H6*B*⋯O5^iv^	0.89 (3)	2.19 (3)	3.025 (2)	156 (3)

Symmetry codes: (i) 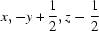; (ii) 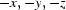; (iii) 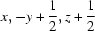; (iv) 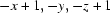.

**Fig. 1.**
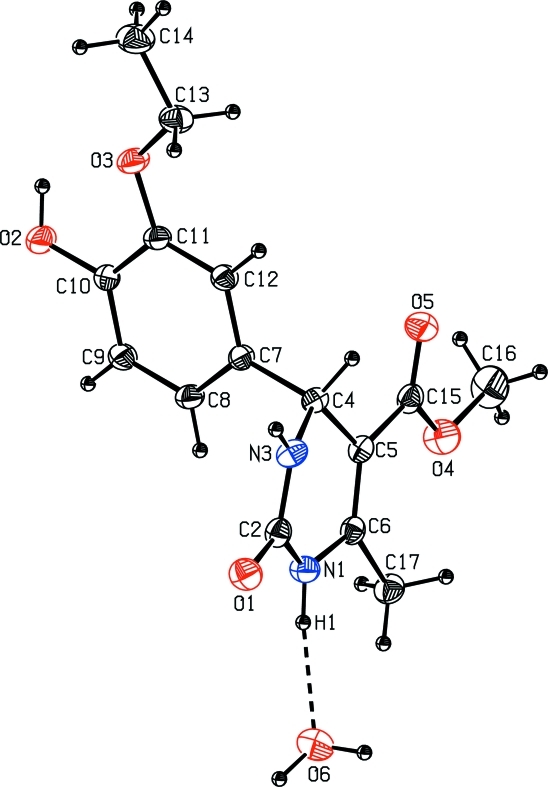
The molecular structure of the title compound, showing 30% probability displacement ellipsoids. The dashed line indicates a hydrogen bond.

**Fig. 2.**
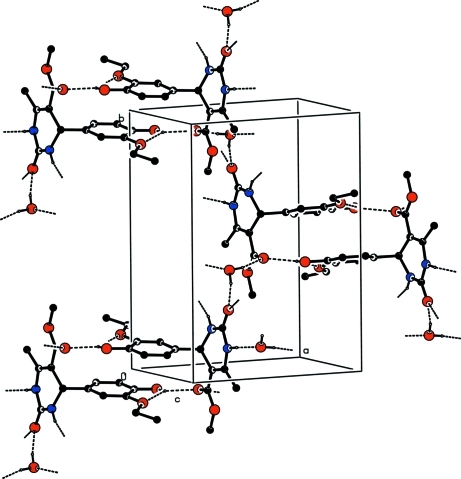
Part of the crystal packing of the title compound, showing hydrogen-bonded (dashed lines) dimers.

## supplementary crystallographic information

### Comment

Pyrimidines are considered to be important not only they form an integral part of the genetic material (*viz*. DNA and RNA), but also impart numerous biological activities such as bactericides, fungicides, viricides, insecticides and meticides. They have also found applications in agricultural and industrial chemicals (Kidwai *et al.*, 2003).

The pyrimidine ring assumes a flattened-boat conformation with puckering parameters (Cremer & Pople, 1975) q_2_ = 0.241 (1) Å, q_3_ = 0.077 (1) Å and φ= 12.5 (3)°, and the asymmetry parameter (Nardelli, 1983) Δ_s_(N1,C4) = 8.4 (2)°. The hydroxyphenyl ring is almost perpendicular to the C2/N3/C5/C6 plane, with a dihedral angle of 85.13 (7)°. The ethoxy [C14—C13—O3—C11 = -177.41 (13)°] and carboxylate [C5—C15—O4—C16 = 179.32 (14)°] groups attached to the pyrimidine ring exhibit extented conformations. The sum of the bond angles around atom N1 [357.4°] of the pyrimidine ring is in accordance with *sp*^2^ hybridization (Beddoes *et al.*, 1986). The C2═O1 bond length of 1.2420 (18) Å is close to the expected value of 1.225 Å for a free, unbridged bond (Litvinov *et al.*, 1992).

The molecules are linked into centrosymmetric *R*_2_^2^(20) dimers by O—H···O hydrogen bonds and the dimers are linked by N—H···O hydrogen bonds to form a two-dimensional network parallel to the *bc* plane. The adjacent networks are cross-linked via N—H···O and O—H···O hydrogen bonds involving the water molecules.

### Experimental

A mixture of 3-ethoxy-4-hydroxy benzaldehyde (10 mmol), methylacetoacetate (12 mmol), urea (15 mmol) and 1 ml of conc. HCl was placed in a round bottom flask containing 30 ml of acetonitrile. The reaction mixture was refluxed for 5 h at 348-353 K. After completion of the reaction (checked by TLC), the reaction mixture was poured in ice cooled water. The separated solid was filtered, dried and recrystallized with methanol.

### Refinement

O- and N-bound H atoms were located in a difference map and refined freely. C-bound H atoms were positioned geometrically (C-H = 0.93–0.98 Å) and allowed to ride on their parent atoms, with *U*_iso_(H) = 1.2–1.5(methyl) *U*_eq_(C).

### Crystal data

**Table:**

C_15_H_18_N_2_O_5_·H_2_O	*F*(000) = 688
*M**_r_* = 324.33	*D*_x_ = 1.373 Mg m^−^^3^
Monoclinic, *P*2_1_/*c*	Mo *K*α radiation, λ = 0.71073 Å
Hall symbol: -P 2ybc	Cell parameters from 4719 reflections
*a* = 11.4927 (6) Å	θ = 1.8–30.3°
*b* = 15.3756 (8) Å	µ = 0.11 mm^−^^1^
*c* = 8.9240 (5) Å	*T* = 293 K
β = 95.932 (2)°	Block, yellow
*V* = 1568.49 (15) Å^3^	0.25 × 0.20 × 0.20 mm
*Z* = 4	

### Data collection

**Table:**

Bruker Kappa APEXII area-detector diffractometer	4719 independent reflections
Radiation source: fine-focus sealed tube	3292 reflections with *I* > 2σ(*I*)
graphite	*R*_int_ = 0.025
ω and φ scans	θ_max_ = 30.3°, θ_min_ = 1.8°
Absorption correction: multi-scan (*SADABS*; Sheldrick, 2001)	*h* = −16→16
*T*_min_ = 0.977, *T*_max_ = 0.981	*k* = −21→21
20671 measured reflections	*l* = −12→12

### Refinement

**Table:**

Refinement on *F*^2^	Primary atom site location: structure-invariant direct methods
Least-squares matrix: full	Secondary atom site location: difference Fourier map
*R*[*F*^2^ > 2σ(*F*^2^)] = 0.048	Hydrogen site location: inferred from neighbouring sites
*wR*(*F*^2^) = 0.140	H atoms treated by a mixture of independent and constrained refinement
*S* = 1.03	*w* = 1/[σ^2^(*F*_o_^2^) + (0.0643*P*)^2^ + 0.381*P*] where *P* = (*F*_o_^2^ + 2*F*_c_^2^)/3
4719 reflections	(Δ/σ)_max_ = 0.001
231 parameters	Δρ_max_ = 0.31 e Å^−^^3^
0 restraints	Δρ_min_ = −0.25 e Å^−^^3^

### Special details

**Table:**

Geometry. All e.s.d.'s (except the e.s.d. in the dihedral angle between two l.s. planes) are estimated using the full covariance matrix. The cell e.s.d.'s are taken into account individually in the estimation of e.s.d.'s in distances, angles and torsion angles; correlations between e.s.d.'s in cell parameters are only used when they are defined by crystal symmetry. An approximate (isotropic) treatment of cell e.s.d.'s is used for estimating e.s.d.'s involving l.s. planes.
Refinement. Refinement of *F*^2^ against ALL reflections. The weighted *R*-factor *wR* and goodness of fit *S* are based on *F*^2^, conventional *R*-factors *R* are based on *F*, with *F* set to zero for negative *F*^2^. The threshold expression of *F*^2^ > σ(*F*^2^) is used only for calculating *R*-factors(gt) *etc*. and is not relevant to the choice of reflections for refinement. *R*-factors based on *F*^2^ are statistically about twice as large as those based on *F*, and *R*- factors based on ALL data will be even larger.

### Fractional atomic coordinates and isotropic or equivalent isotropic displacement parameters (Å^2^)

**Table:**

	*x*	*y*	*z*	*U*_iso_*/*U*_eq_	
C2	0.35042 (12)	0.17029 (9)	0.52062 (17)	0.0356 (3)	
C4	0.30165 (12)	0.07826 (8)	0.29487 (15)	0.0323 (3)	
H4	0.3447	0.0720	0.2063	0.039*	
C5	0.33233 (11)	0.00144 (8)	0.39770 (15)	0.0318 (3)	
C6	0.35124 (11)	0.01266 (8)	0.54780 (16)	0.0321 (3)	
C7	0.17205 (12)	0.08179 (8)	0.24074 (15)	0.0314 (3)	
C8	0.08815 (12)	0.06691 (10)	0.33778 (15)	0.0372 (3)	
H8	0.1109	0.0543	0.4385	0.045*	
C9	−0.02997 (13)	0.07060 (10)	0.28638 (16)	0.0394 (3)	
H9	−0.0858	0.0606	0.3528	0.047*	
C10	−0.06474 (12)	0.08894 (9)	0.13824 (15)	0.0347 (3)	
C11	0.01924 (12)	0.10656 (9)	0.03979 (15)	0.0329 (3)	
C12	0.13663 (12)	0.10213 (9)	0.09088 (15)	0.0337 (3)	
H12	0.1925	0.1128	0.0248	0.040*	
C13	0.05268 (15)	0.15592 (11)	−0.20597 (17)	0.0458 (4)	
H13A	0.1074	0.1099	−0.2233	0.055*	
H13B	0.0966	0.2062	−0.1664	0.055*	
C14	−0.01997 (19)	0.17865 (13)	−0.34907 (19)	0.0579 (5)	
H14A	−0.0642	0.1287	−0.3857	0.087*	
H14B	0.0301	0.1968	−0.4229	0.087*	
H14C	−0.0725	0.2251	−0.3306	0.087*	
C15	0.33489 (12)	−0.08120 (9)	0.31629 (17)	0.0373 (3)	
C16	0.3448 (2)	−0.23424 (11)	0.3223 (3)	0.0685 (6)	
H16A	0.2807	−0.2358	0.2442	0.103*	
H16B	0.3372	−0.2813	0.3913	0.103*	
H16C	0.4172	−0.2400	0.2783	0.103*	
C17	0.37615 (14)	−0.05489 (10)	0.66793 (17)	0.0422 (3)	
H17A	0.3058	−0.0866	0.6803	0.063*	
H17B	0.4037	−0.0271	0.7612	0.063*	
H17C	0.4348	−0.0942	0.6392	0.063*	
N1	0.34801 (11)	0.09613 (8)	0.60651 (15)	0.0381 (3)	
N3	0.34091 (11)	0.15896 (8)	0.37262 (14)	0.0371 (3)	
O1	0.36409 (10)	0.24219 (7)	0.58341 (13)	0.0474 (3)	
O2	−0.18113 (9)	0.09133 (9)	0.09076 (14)	0.0498 (3)	
O3	−0.02656 (9)	0.12778 (8)	−0.10273 (11)	0.0444 (3)	
O4	0.34354 (12)	−0.15218 (7)	0.40215 (14)	0.0555 (3)	
O5	0.32921 (11)	−0.08496 (8)	0.18008 (13)	0.0521 (3)	
O6	0.43101 (15)	0.11524 (11)	0.92163 (16)	0.0654 (4)	
H1	0.3666 (16)	0.1039 (11)	0.703 (2)	0.045 (5)*	
H2	−0.193 (2)	0.0937 (15)	−0.010 (3)	0.077 (7)*	
H3	0.3418 (15)	0.2040 (12)	0.317 (2)	0.048 (5)*	
H6A	0.418 (2)	0.1623 (17)	0.981 (3)	0.086 (8)*	
H6B	0.508 (3)	0.109 (2)	0.921 (4)	0.136 (13)*	

### Atomic displacement parameters (Å^2^)

**Table:**

	*U*^11^	*U*^22^	*U*^33^	*U*^12^	*U*^13^	*U*^23^
C2	0.0302 (7)	0.0344 (6)	0.0417 (8)	−0.0002 (5)	0.0015 (6)	−0.0002 (5)
C4	0.0312 (6)	0.0350 (6)	0.0301 (7)	−0.0004 (5)	0.0001 (5)	0.0019 (5)
C5	0.0280 (6)	0.0315 (6)	0.0353 (7)	0.0000 (5)	0.0006 (5)	0.0025 (5)
C6	0.0263 (6)	0.0343 (6)	0.0360 (7)	0.0002 (5)	0.0043 (5)	0.0037 (5)
C7	0.0310 (6)	0.0325 (6)	0.0301 (6)	0.0003 (5)	0.0013 (5)	0.0003 (5)
C8	0.0363 (7)	0.0490 (8)	0.0259 (6)	0.0012 (6)	0.0015 (5)	0.0037 (5)
C9	0.0342 (7)	0.0546 (8)	0.0304 (7)	−0.0001 (6)	0.0079 (5)	0.0020 (6)
C10	0.0288 (7)	0.0439 (7)	0.0313 (7)	0.0014 (5)	0.0028 (5)	−0.0031 (5)
C11	0.0350 (7)	0.0379 (7)	0.0254 (6)	0.0002 (5)	0.0010 (5)	0.0012 (5)
C12	0.0322 (7)	0.0400 (7)	0.0294 (6)	−0.0016 (5)	0.0055 (5)	0.0021 (5)
C13	0.0518 (9)	0.0531 (9)	0.0332 (8)	−0.0040 (7)	0.0082 (7)	0.0053 (6)
C14	0.0819 (14)	0.0552 (10)	0.0366 (9)	0.0059 (9)	0.0054 (9)	0.0085 (7)
C15	0.0290 (7)	0.0372 (7)	0.0445 (8)	0.0001 (5)	−0.0020 (6)	−0.0031 (6)
C16	0.0751 (13)	0.0344 (8)	0.0933 (16)	−0.0003 (8)	−0.0045 (11)	−0.0136 (9)
C17	0.0440 (8)	0.0438 (8)	0.0388 (8)	0.0028 (6)	0.0050 (6)	0.0113 (6)
N1	0.0443 (7)	0.0375 (6)	0.0323 (6)	0.0001 (5)	0.0028 (5)	0.0002 (5)
N3	0.0406 (7)	0.0319 (6)	0.0373 (6)	−0.0047 (5)	−0.0026 (5)	0.0063 (5)
O1	0.0557 (7)	0.0356 (5)	0.0501 (7)	−0.0013 (5)	0.0018 (5)	−0.0059 (4)
O2	0.0289 (5)	0.0835 (9)	0.0366 (6)	0.0018 (5)	0.0019 (4)	−0.0008 (6)
O3	0.0380 (6)	0.0664 (7)	0.0281 (5)	−0.0014 (5)	0.0005 (4)	0.0087 (5)
O4	0.0748 (9)	0.0310 (5)	0.0591 (8)	0.0006 (5)	−0.0011 (6)	−0.0010 (5)
O5	0.0593 (7)	0.0524 (7)	0.0431 (7)	0.0062 (5)	−0.0016 (5)	−0.0114 (5)
O6	0.0703 (10)	0.0753 (9)	0.0500 (8)	0.0054 (8)	0.0034 (7)	−0.0158 (7)

### Geometric parameters (Å, °)

**Table:**

C2—O1	1.2421 (16)	C13—O3	1.4279 (19)
C2—N3	1.3255 (19)	C13—C14	1.493 (2)
C2—N1	1.3758 (18)	C13—H13A	0.97
C4—N3	1.4696 (17)	C13—H13B	0.97
C4—C5	1.5149 (18)	C14—H14A	0.96
C4—C7	1.5186 (18)	C14—H14B	0.96
C4—H4	0.98	C14—H14C	0.96
C5—C6	1.3457 (19)	C15—O5	1.2119 (18)
C5—C15	1.4654 (19)	C15—O4	1.3314 (18)
C6—N1	1.3881 (18)	C16—O4	1.450 (2)
C6—C17	1.4988 (19)	C16—H16A	0.96
C7—C8	1.3801 (19)	C16—H16B	0.96
C7—C12	1.3926 (18)	C16—H16C	0.96
C8—C9	1.388 (2)	C17—H17A	0.96
C8—H8	0.93	C17—H17B	0.96
C9—C10	1.370 (2)	C17—H17C	0.96
C9—H9	0.93	N1—H1	0.877 (19)
C10—O2	1.3613 (17)	N3—H3	0.852 (19)
C10—C11	1.3972 (19)	O2—H2	0.90 (2)
C11—O3	1.3648 (16)	O6—H6A	0.92 (3)
C11—C12	1.3804 (19)	O6—H6B	0.89 (4)
C12—H12	0.93		
			
O1—C2—N3	124.06 (13)	C14—C13—H13A	110.4
O1—C2—N1	119.69 (14)	O3—C13—H13B	110.4
N3—C2—N1	116.23 (12)	C14—C13—H13B	110.4
N3—C4—C5	109.36 (11)	H13A—C13—H13B	108.6
N3—C4—C7	111.30 (11)	C13—C14—H14A	109.5
C5—C4—C7	112.32 (11)	C13—C14—H14B	109.5
N3—C4—H4	107.9	H14A—C14—H14B	109.5
C5—C4—H4	107.9	C13—C14—H14C	109.5
C7—C4—H4	107.9	H14A—C14—H14C	109.5
C6—C5—C15	126.53 (12)	H14B—C14—H14C	109.5
C6—C5—C4	120.43 (12)	O5—C15—O4	122.09 (13)
C15—C5—C4	113.02 (12)	O5—C15—C5	122.48 (13)
C5—C6—N1	119.10 (12)	O4—C15—C5	115.44 (13)
C5—C6—C17	128.48 (13)	O4—C16—H16A	109.5
N1—C6—C17	112.41 (12)	O4—C16—H16B	109.5
C8—C7—C12	119.08 (12)	H16A—C16—H16B	109.5
C8—C7—C4	121.36 (12)	O4—C16—H16C	109.5
C12—C7—C4	119.55 (12)	H16A—C16—H16C	109.5
C7—C8—C9	120.57 (13)	H16B—C16—H16C	109.5
C7—C8—H8	119.7	C6—C17—H17A	109.5
C9—C8—H8	119.7	C6—C17—H17B	109.5
C10—C9—C8	120.32 (13)	H17A—C17—H17B	109.5
C10—C9—H9	119.8	C6—C17—H17C	109.5
C8—C9—H9	119.8	H17A—C17—H17C	109.5
O2—C10—C9	119.01 (13)	H17B—C17—H17C	109.5
O2—C10—C11	121.30 (12)	C2—N1—C6	123.58 (13)
C9—C10—C11	119.67 (13)	C2—N1—H1	114.8 (12)
O3—C11—C12	126.10 (12)	C6—N1—H1	119.0 (12)
O3—C11—C10	114.04 (12)	C2—N3—C4	124.82 (12)
C12—C11—C10	119.86 (12)	C2—N3—H3	118.0 (12)
C11—C12—C7	120.46 (12)	C4—N3—H3	115.6 (12)
C11—C12—H12	119.8	C10—O2—H2	111.0 (15)
C7—C12—H12	119.8	C11—O3—C13	117.61 (12)
O3—C13—C14	106.57 (14)	C15—O4—C16	115.74 (14)
O3—C13—H13A	110.4	H6A—O6—H6B	108 (3)
			
N3—C4—C5—C6	21.65 (17)	O3—C11—C12—C7	178.32 (13)
C7—C4—C5—C6	−102.44 (14)	C10—C11—C12—C7	−1.2 (2)
N3—C4—C5—C15	−159.91 (11)	C8—C7—C12—C11	−0.6 (2)
C7—C4—C5—C15	76.00 (14)	C4—C7—C12—C11	−179.32 (12)
C15—C5—C6—N1	178.59 (12)	C6—C5—C15—O5	−171.98 (14)
C4—C5—C6—N1	−3.19 (19)	C4—C5—C15—O5	9.7 (2)
C15—C5—C6—C17	−1.7 (2)	C6—C5—C15—O4	8.2 (2)
C4—C5—C6—C17	176.53 (13)	C4—C5—C15—O4	−170.10 (12)
N3—C4—C7—C8	−79.50 (16)	O1—C2—N1—C6	−169.74 (13)
C5—C4—C7—C8	43.50 (17)	N3—C2—N1—C6	8.4 (2)
N3—C4—C7—C12	99.20 (14)	C5—C6—N1—C2	−13.8 (2)
C5—C4—C7—C12	−137.80 (13)	C17—C6—N1—C2	166.38 (13)
C12—C7—C8—C9	1.1 (2)	O1—C2—N3—C4	−167.13 (14)
C4—C7—C8—C9	179.85 (13)	N1—C2—N3—C4	14.8 (2)
C7—C8—C9—C10	0.1 (2)	C5—C4—N3—C2	−28.53 (18)
C8—C9—C10—O2	179.40 (14)	C7—C4—N3—C2	96.16 (15)
C8—C9—C10—C11	−1.9 (2)	C12—C11—O3—C13	−6.2 (2)
O2—C10—C11—O3	1.5 (2)	C10—C11—O3—C13	173.34 (13)
C9—C10—C11—O3	−177.11 (13)	C14—C13—O3—C11	−177.41 (13)
O2—C10—C11—C12	−178.89 (13)	O5—C15—O4—C16	−0.5 (2)
C9—C10—C11—C12	2.5 (2)	C5—C15—O4—C16	179.32 (14)

### Hydrogen-bond geometry (Å, °)

**Table:**

*D*—H···*A*	*D*—H	H···*A*	*D*···*A*	*D*—H···*A*
N1—H1···O6	0.87 (2)	2.02 (2)	2.890 (2)	172 (2)
N3—H3···O1^i^	0.85 (2)	2.28 (2)	3.031 (2)	147 (2)
O2—H2···O5^ii^	0.90 (2)	2.07 (2)	2.810 (2)	139 (2)
O6—H6A···O1^iii^	0.92 (3)	1.87 (3)	2.777 (2)	170 (2)
O6—H6B···O5^iv^	0.89 (3)	2.19 (3)	3.025 (2)	156 (3)

Symmetry codes: (i) *x*, −*y*+1/2, *z*−1/2; (ii) −*x*, −*y*, −*z*; (iii) *x*, −*y*+1/2, *z*+1/2; (iv) −*x*+1, −*y*, −*z*+1.
